# Evaluation of a real-time PCR assay for detection and quantification of bacterial DNA directly in blood of preterm neonates with suspected late-onset sepsis

**DOI:** 10.1186/s13054-018-2010-4

**Published:** 2018-04-22

**Authors:** Marre van den Brand, Frank A. M. van den Dungen, Martine P. Bos, Mirjam M. van Weissenbruch, A. Marceline van Furth, Annemieke de Lange, Anna Rubenjan, Remco P. H. Peters, Paul H. M. Savelkoul

**Affiliations:** 1 Department of Medical Microbiology and Infection Control, VU University Medical Center, De Boelelaan 1117, 1081HV Amsterdam, The Netherlands; 2 Department of Pediatrics, VU University Medical Center, De Boelelaan 1117, 1081HV Amsterdam, The Netherlands; 3 Microbiome Ltd., De Boelelaan 1108, 1081HZ Amsterdam, The Netherlands; 4 Department of Medical Microbiology, University of Pretoria, Lynnwood Road and Roper Street, Hatfield, South Africa; 5 Department of Medical Microbiology, Maastricht University Medical Centre+, P. Debyelaan 25, 6229HX Maastricht, The Netherlands

**Keywords:** Molecular diagnosis, Late-onset sepsis, Neonatology, Real-time PCR, Bacterial DNA load, Bacteremia

## Abstract

**Background:**

Rapid and accurate diagnosis of neonatal sepsis is highly warranted because of high associated morbidity and mortality. The aim of this study was to evaluate the performance of a novel multiplex PCR assay for diagnosis of late-onset sepsis and to investigate the value of bacterial DNA load (BDL) determination as a measure of infection severity.

**Methods:**

This cross-sectional study was conducted in a neonatal intensive care unit. Preterm and/or very low birth weight infants suspected for late-onset sepsis were included. Upon suspicion of sepsis, a whole blood sample was drawn for multiplex PCR to detect the eight most common bacteria causing neonatal sepsis, as well as for blood culture. BDL was determined in episodes with a positive multiplex PCR.

**Results:**

In total, 91 episodes of suspected sepsis were investigated, and PCR was positive in 53 (58%) and blood culture in 60 (66%) episodes, yielding no significant difference in detection rate (*p* = 0.17). Multiplex PCR showed a sensitivity of 77%, specificity of 81%, positive predictive value of 87%, and negative predictive value of 68% compared with blood culture. Episodes with discordant results of PCR and blood culture included mainly detection of coagulase-negative staphylococci (CoNS). C-reactive protein (CRP) level and immature to total neutrophil (I/T) ratio were lower in these episodes, indicating less severe disease or even contamination. Median BDL was high (4.1 log_10_ cfu Eq/ml) with a wide range, and was it higher in episodes with a positive blood culture than in those with a negative blood culture (4.5 versus 2.5 log_10_ cfu Eq/ml; *p* < 0.0001). For CoNS infection episodes BDL and CRP were positively associated (*p* = 0.004), and for *Staphylococcus aureus* infection episodes there was a positive association between BDL and I/T ratio (*p* = 0.049).

**Conclusions:**

Multiplex PCR provides a powerful assay to enhance rapid identification of the causative pathogen in late-onset sepsis. BDL measurement may be a useful indicator of severity of infection.

**Electronic supplementary material:**

The online version of this article (10.1186/s13054-018-2010-4) contains supplementary material, which is available to authorized users.

## Background

Late-onset sepsis (LOS) is a serious nosocomial infection common among preterm and very low birth weight (VLBW) infants. Over 60% of VLBW infants are suspected of LOS at some point during hospitalization, and LOS is confirmed through blood culture in one-third of these infants [1]. Compared with infants without LOS, infants with LOS have a higher mortality rate, require longer hospitalization, and are at increased risk of impaired neurodevelopmental outcome [1–3]. Because most deaths occur in the first days following the onset of symptoms, accurate and rapid diagnosis followed by the initiation of appropriate antibiotic therapy is of great importance.

Diagnosing LOS clinically can be challenging because signs and symptoms are generally nonspecific. Furthermore, blood culture, the diagnostic gold standard, often remains negative owing to the small blood volume that can be obtained from neonates and/or because of prior use of antibiotics [4, 5]. The clinical impact of the blood culture is also negatively affected by its turnaround time (TAT) because it usually requires 1 day of incubation. Several biomarkers have been identified as useful, rapid tools to aid in the diagnosis of LOS, but they fail to provide information on the causative pathogen [6, 7]. As a result, the development of a diagnostic test that detects pathogens that cause LOS in a rapid, sensitive, and specific manner is highly warranted to overcome the current limitations in diagnosing LOS.

Over the past decade, researchers in several studies have reported the use of real-time PCR for diagnosis of neonatal sepsis, and a recent meta-analysis indicates that PCR may be a valuable tool in the diagnostic arsenal [8–12]. For example, Jordan et al. have conducted several studies using broad-range 16S ribosomal RNA gene PCR to rapidly diagnose sepsis in a low-risk neonatal population [11, 13, 14]. Researchers in another interesting study successfully used a gram-specific PCR to distinguish between gram-positive and gram-negative sepsis in order to anticipate the more severe course of gram-negative sepsis [12]. Although these PCR assay results are promising, widespread implementation in routine practice has not yet occurred, because these assays require additional steps to identify the pathogen to the species level, which is costly and labor-intensive and prolongs the TAT. Multiplex real-time PCR could overcome this issue, but it has been used in LOS only sporadically [15, 16]. We recently reported on a multiplex assay that rapidly identifies the most common pathogens that cause LOS using real-time PCR [17].

Another advantage of real-time PCR is that it allows quantitative measurements and thereby enables the determination of the bacterial DNA load (BDL) [18]. A growing body of evidence indicates a relationship between the BDL measured during infection and severity of disease [19–24]. For example, for meningococcal disease, Darton et al. demonstrated an association between a high BDL and an increased incidence of renal failure and death [20]. To our knowledge, the BDL has not been studied in a species-specific manner in infants with LOS. Besides providing potentially crucial information on the severity of disease in LOS, BDL may help to distinguish true infection from contamination in the specific case of coagulase-negative staphylococci (CoNS).

In this study, we aimed to evaluate the clinical utility of species-specific detection of bacterial DNA in whole blood samples for the diagnosis of LOS in preterm infants. Also, we evaluated the value of BDL in assessing the severity of infection.

## Methods

### Study population

This study was a cross-sectional analysis of baseline data collected in a prospective study that was conducted in the neonatal intensive care unit of the VU University Medical Center, Amsterdam, between March 2009 and July 2013. Preterm (gestational age < 32 weeks) as well as VLBW (< 1500 g) infants suspected of having a nosocomial (age at onset > 72 h and hospitalized) bloodstream infection were eligible for the study. Patients with a (suspected) genetic or metabolic disorder were excluded from participation. The Medical Ethics Review Committee of the VU University Medical Center (reference 2008/77) approved the study, and written informed consent was obtained from the parents or legal guardians of all neonates prior to inclusion. The study protocol allowed the inclusion of multiple episodes of suspected nosocomial sepsis occurring in one infant.

### Sample collection

Upon clinical suspicion of a bloodstream infection, before initiation of antibiotic treatment, a whole blood sample was collected aseptically through a newly inserted catheter for blood culture (BD BACTEC Peds Plus™/F Medium; BD Biosciences, San Jose, CA, USA), PCR, and infection parameters. In a few episodes, an arterial (peripheral or umbilical) line was present and used for blood sampling. Blood samples for PCR were stored at −80 °C until further processing. Additional body samples for culture (e.g., urine, cerebrospinal fluid, tracheal aspirate, pustule) were obtained on indication of the physician. Blood cultures were incubated for 5 days (BD BACTEC 9240; BD Biosciences), and in case of a positive (blood) culture, microorganisms were identified using standard laboratory procedures, including matrix-assisted laser desorption ionization-time of flight mass spectrometry with the VITEK MS system (bioMérieux, Zaltbommel, The Netherlands). The length of time that the blood culture spent in the BACTEC incubator was recorded as the time to positivity.

### DNA isolation and multiplex PCR assay

The design and validation of the multiplex real-time PCR assay, as well as the DNA isolation process, are described in detail elsewhere [17]. Briefly, DNA was isolated from 200 μl of whole blood with bacterial lysis buffer (Biocartis, Mechelen, Belgium) and the NucliSENS easyMag device (bioMérieux). Prior to DNA purification, samples were spiked with phocine herpesvirus 1 as extraction and amplification control (EAC). Positive and negative process controls were included.

**Fig. 1:**
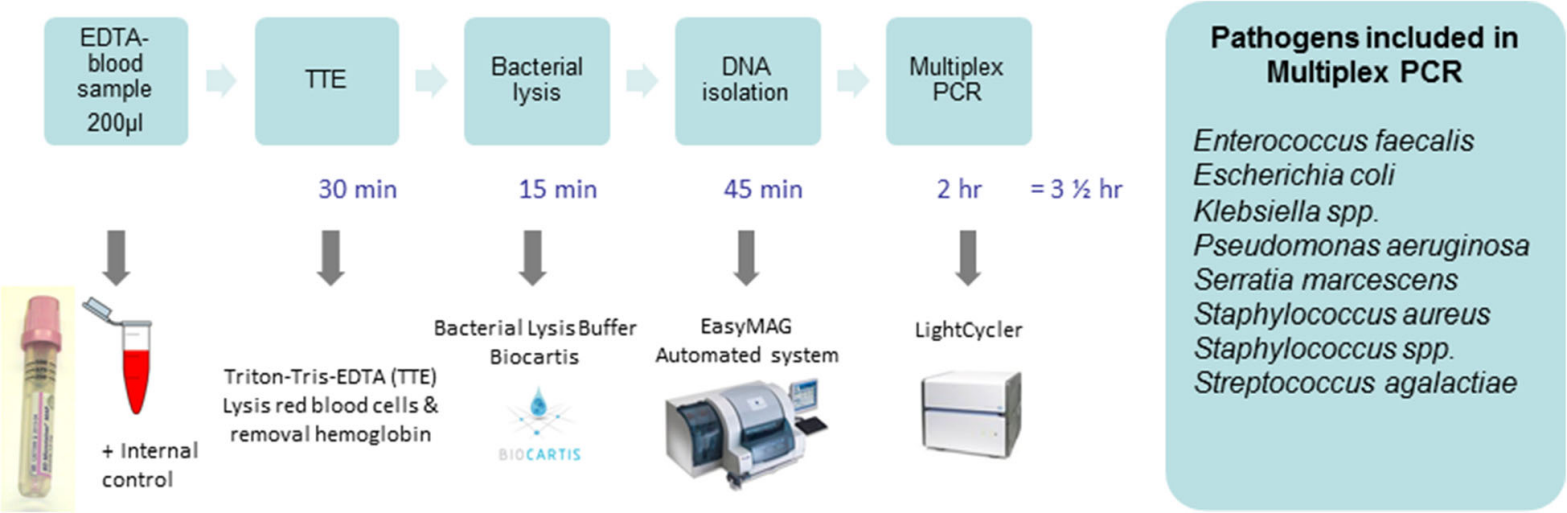
Sample processing method and multiplex PCR assay. *EDTA* Ethylenediaminetetraacetic acid

The multiplex PCR assay, which consists of three reactions, allows the detection of the eight most common bacterial pathogens of LOS directly from blood in amounts as small as ten copies/PCR (Fig. 1) [17]. The PCRs were performed on a LightCycler 480 Instrument II (Roche Diagnostics, Almere, The Netherlands). If amplification was observed, the sample was subsequently tested in a monoplex reaction for BDL determination as described below.

### Interpretation of PCR results and determination of BDL

PCR results were analyzed blinded for blood culture results. A sample was regarded as negative when it was noninhibited and all pathogen PCRs were negative. The sample was considered noninhibited when the quantification cycle (C_q_) value of the EAC signal of the sample was within ± 2 SD of the EAC signal in the negative control samples.

Because the *Staphylococcus spp.* PCRs occasionally yielded low positive signals in negative control samples, a cutoff was set for the clinical samples: These were regarded as positive only in case of a C_q_ value ≤ 39 with fluorescence ≥ 50,000 arbitrary units and ≥ 2 C_q_ values lower than observed in the negative control sample. A sample was regarded to be positive for both CoNS and *Staphylococcus aureus* if the C_q_ value of the *Staphylococcus spp.* assay was 3 C_q_ values lower than the *S. aureus*-specific assay. In the *S. aureus*-specific assay, low amplification signals were confirmed with a second PCR assay.

The BDL was determined by including tenfold serial dilutions of cloned PCR amplicons to serve as a standard curve. The corresponding BDL expressed in log_10_ cfu Eq/ml was determined by correcting for blood volume and number of PCR target copies per genome.

### Clinical data and definitions

Data were extracted from the patients’ medical records and included demographic, laboratory, and clinical data on the suspected infectious episode and outcome. The leukocyte count was further specified by calculating the immature to total neutrophil (I/T) ratio [25]. Episodes were included when clinical suspicion of LOS occurred in an infant who did not receive systemic antibiotics at least 24 h prior to onset of symptoms. Culture-proven sepsis was defined as an episode with at least one positive blood culture with a microorganism not considered as a contaminant (e.g., *Bacillus spp.*), and targeted antibiotic treatment continued for ≥ 7 days or until removal of incriminated vascular access. Clinical sepsis was defined as above but with negative blood culture(s). Localized infection was defined as an episode with clinical signs consistent with localized infection and a positive culture from a relevant body site or positive imaging in the absence of a positive blood culture and if targeted treatment continued for ≥ 7 days. No infection was defined as an episode with negative cultures and in which treatment was discontinued after 48–72 h. A polymicrobial infection episode was defined as an episode in which more than one microorganism was detected by either multiplex PCR or blood culture.

### Statistical analysis

Statistical analysis was performed with IBM SPSS Statistics version 22.0 software (IBM, Armonk, NY, USA)**.** Sensitivity, specificity, positive predictive value, and negative predictive value were calculated with standard two-by-two tables and blood culture as the gold standard. The difference in positivity rate between blood culture and PCR was explored with the McNemar test. Comparison of groups was performed using the Mann-Whitney *U* test for continuous variables and the chi-square test or Fisher’s exact test for categorical variables. Spearman’s rank correlation was applied to investigate the relationship between continuous variables. Subsequently, linear (hierarchical) regression analysis was performed if applicable.

## Results

### Study population

In total, 91 episodes of suspected LOS occurring in 77 neonates were included, of which 13 infants contributed 2 episodes and 1 infant 3 episodes of suspected LOS **(**Table 1**)**. Culture-proven sepsis was diagnosed in 57 episodes (63%), clinical sepsis in 4 (4%), and localized infection in 9 (10%), whereas no infection was diagnosed in 21 episodes (23%). Localized infection included five episodes of necrotizing enterocolitis, three episodes of pneumonia, and one episode of urinary tract infection.

**Table 1:** Summary of study population

Characteristics	Data
Characteristics of neonates^a^ (*N* = 77)	
Sex, male	39 (51)
Gestational age, weeks	28.1 (26.5–29.5)
Birth weight, g	1080 (775–1235)
Duration of hospitalization, days	33 (20–53)
Outcome, died	8 (10)
Clinical characteristics of included episodes (*N* = 91)	
Age at onset of symptoms, days	10 (7–15)
Weight at onset of symptoms, g	1087 (870–1310)^b^
CRP (mg/L)	12 (2.4–34)^c^
White blood cell count (×10^9^/L)	13.2 (7.7–20.6)^c^
Blood culture positive	60 (66)
CoNS	47
*Staphylococcus aureus*	11
*Streptococcus agalactiae*	1
*Enterococcus faecalis*	1
*Escherichia coli*	2
*Klebsiella spp.*	1
*Lactobacillus spp.*	1
Cerebrospinal fluid, obtained/positive	51/5 (6)
Polymicrobial infection	7 (8)
Duration of antibiotic treatment, days	7 (3–11)
Diagnosis	
Culture-proven sepsis	57 (63)
Clinical sepsis	4 (4)
Localized infection	9 (10)
No infection	21 (23)
Blood culture TTP	14 (11–19)

*Abbreviations: CoNS* Coagulase-negative staphylococci, *CRP* C-reactive protein, *TTP* Time to positivity

^a^Continuous variables are presented as median (25–75% interquartile range). Categorical variables are presented as number (percent)

^b^Data missing for two infection episodes

^c^Data missing for one infection episode

### Evaluation of multiplex PCR for diagnosis of neonatal sepsis

Multiplex PCR was positive in 53 episodes (58%) and blood culture in 60 (66%). Blood culture and PCR were concordant positive in 41 episodes, concordant negative in 25, and discordant in 25. Six discordant episodes were detected by PCR only, 12 by blood culture only, and 7 were polymicrobial (Table 2**;** Additional file 1). There was no significant difference in positivity rates between both assays (*p* = 0.17). CoNS were the most frequently detected microorganisms, followed by *S. aureus* (Table 2). *Streptococcus agalactiae* was detected in three episodes (two episodes detected only by PCR). Gram-negative sepsis was rare (*n* = 4), and *Pseudomonas aeruginosa* and *Serratia marcescens* were not detected by any method.

**Table 2:** Performance of the multiplex PCR assay compared with blood culture (polymicrobial infections excluded)

		Blood culture		Total
		+	–	
PCR	+	31 CoNS 7 *Staphylococcus aureus* 2 *Escherichia coli* 1 *Streptococcus agalactiae*	4 CoNS 1 *S. aureus* 1 *S. agalactiae*	47
	–	11 CoNS 1 *S. aureus*	25	37
Total		53	31	84

For infection episodes (*n* = 84, excluding polymicrobial infections) the multiplex PCR demonstrated a sensitivity of 77% (95% CI 64–88), specificity of 81% (95% CI 63–93), positive predictive value of 87% (95% CI 74–95), and negative predictive value of 68% (95% CI 50–82) compared with blood culture. In this study, seven episodes were polymicrobial, of which three were detected by PCR only and two by blood culture only (*see* Additional file 1). All episodes with polymicrobial infections showed discordant results between blood culture and PCR, indicating the difficulty of diagnosing polymicrobial infections. For example, for one infant with necrotizing enterocolitis, blood culture grew lactobacilli, whereas PCR detected *S. agalactiae, Enterococcus faecalis, Klebsiella spp.,* and CoNS.

### Evaluation of patients with discordant results between blood culture and PCR

The results of PCR were negative for 11 episodes with CoNS-positive blood culture and 1 with *S. aureus* **(**Table 2**)**. Ten of these episodes were considered culture-proven sepsis, whereas in two episodes, blood culture was regarded to be contaminated **(**Table 3**)**. Therefore, the PCR results were presumably false-negative in ten episodes and true-negative in two episodes (*for details*, *see* Table 3, patients 5 and 12**)**.

**Table 3:** Details of episodes of suspected sepsis with discordant results for blood culture and PCR

Patient	Blood culture (+/−)/PCR (+/−)	Blood culture microorganism	PCR microorganism	BDL cfu Eq/ml	Culture of other sites^a^ (result + microorganism)	CRP (mg/L)	Duration of treatment (days)	Clinical diagnosis	Comments
1	+/−	CoNS	Negative	NA	Tracheal aspirate (*Ureaplasma urealyticum* + *Mycoplasma hominis*)	< 2.5	6	Culture-proven sepsis with CoNS	
2	+/−	CoNS	Negative	NA	CSF (negative) Urine (negative)	< 2.5	6	Culture-proven sepsis with CoNS	
3	+/−	CoNS	Negative	NA	Blood culture *obtained from other site* (negative) Urine (negative)	< 2.5	7	Culture-proven sepsis with CoNS	
4	+/−	CoNS	Negative	NA	Blood culture (CoNS) CSF (negative) Urine (negative)	< 2.5	21	Culture-proven sepsis with CoNS	
5	+/−	CoNS	Negative	NA	CSF (negative) Urine (negative)	< 2.5	2	No infection	
6	+/−	CoNS	Negative	NA	CSF (negative) Urine (negative)	6	7	Culture-proven sepsis with CoNS	
7	+/−	CoNS	Negative	NA	CSF (negative) Urine (CoNS)	4	6	Culture-proven sepsis with CoNS	
8	+/−	CoNS	Negative	NA	CSF (negative) Urine (*Staphylococcus aureus* < 10^3^ + CoNS 10^3^)	< 2.5	6	Culture-proven sepsis with CoNS	
9	+/−	CoNS	Negative	NA	CSF (negative) Skin (*Candida albicans)*	< 2.5	9	Culture-proven sepsis with CoNS	
10	+/−	CoNS	Negative	NA	CSF (negative) PCR (negative)	9	10	Culture-proven sepsis with CoNS	
11	+/−	*S. aureus*	Negative	NA	Pus (*S. aureus*)	85	13	Culture-proven sepsis with *S. aureus* + necrotizing enterocolitis	
12	+/−	CoNS	Negative	NA	Blood culture *obtained from other site* (negative) Tracheal aspirate (*Acinetobacter spp.*)	8	3	No infection	
13	−/+	Negative	CoNS	120	Blood culture (CoNS) CSF (negative) Urine (negative)	47	7	Culture-proven sepsis with CoNS	
14	−/+	Negative	*S. aureus*	Negative	CSF (negative)	9	2	No infection	
15	−/+	Negative	CoNS	2585	Tracheal aspirate (*Ureaplasma spp.*)	< 2.5	2	No infection	Infant showed clinical signs of infection and umbilical catheters were removed
16	−/+	Negative	*Streptococcus agalactiae*	92	Not obtained	86	14	Necrotizing enterocolitis (based on feeding problems and imaging)	*S. agalactiae* is not a usual pathogen in necrotizing enterocolitis but is described in literature [43]
17	−/+	Negative	CoNS	Negative	CSF (negative) Tracheal aspirate (*S. aureus* + *Enterococcus faecalis)* Urine (negative)	16	8	Pneumonia with *S. aureus* (based on positive sputum culture and breathing difficulties requiring intubation)	Two weeks earlier, positive blood culture with CoNS treated with vancomycin for 7 days
18	−/+	Negative	CoNS	2255	CSF (negative) Urine (negative)	< 2.5	2	No infection	

*Abbreviations: BDL* Bacterial DNA load, *CoNS* Coagulase-negative staphylococci, *CRP* C-reactive protein, *CSF* Cerebrospinal fluid, *NA* Not applicable

^a^Blood cultures taken at another site and later point in time are also depicted in this column

Interestingly, multiplex PCR was positive in six episodes associated with a negative blood culture (one *S. aureus*, one *S. agalactiae,* and four CoNS). In two of these episodes, septicemia was very likely based on clinical data and other cultures **(**Table 3, patients 13 and 16**).** In one episode, the PCR result was possibly clinically relevant (patient 17), and in three episodes, its clinical relevance was uncertain.

In episodes with discordant results between blood culture and PCR (*n* = 18), C-reactive protein (CRP) was significantly lower than in episodes with both a positive PCR and blood culture (3 mg/L versus 16 mg/L; *p* = 0.013). The absolute leukocyte count was not different (*p* = 0.99) between these groups, but the I/T ratio was lower in episodes with discordant results than in episodes with concordant positive results (0.07 versus 0.18; *p* = 0.001).

### BDL at onset of neonatal sepsis

**Fig. 2:**
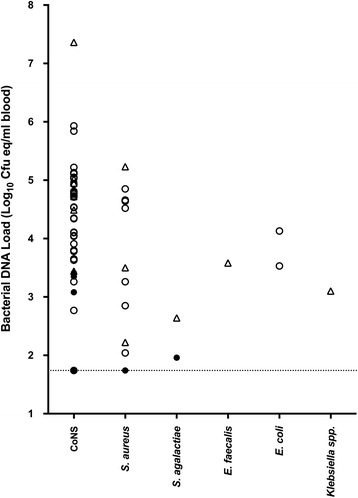
Distribution of the bacterial DNA load at onset of sepsis, stratified by pathogen. Monomicrobial (*circles*) and polymicrobial (*triangles*) infection episodes and blood culture results (*open symbols* = positive, *closed symbols* = negative) are presented as indicated. The *dashed line* indicates the lower detection limit

The BDL was determined for all (*n* = 53) PCR-positive infection episodes, including six polymicrobial infections. Load determination failed in two samples. BDL ranged from 55 to 22,976,150 cfu Eq/ml (median 13.514 cfu Eq/ml; 25–75% interquartile range 2420–63,745 cfu Eq/ml), or from 1.74 to 7.36 log_10_ cfu Eq/ml (median 4.13 log_10_ cfu Eq/ml) (Fig. 2). The BDL was significantly higher in blood culture-positive samples, with a median BDL of 4.5 versus 2.5 log_10_ cfu Eq/ml (*p* < 0.0001). Likewise, BDL and blood culture time to positivity showed a significant association when we took into account the time that had transpired between blood culture collection and incubation at the laboratory (R-square change 0.22; *p* = 0.001).

BDL was evaluated as a marker of severity of infection by investigating the correlation with established markers of infection. For CoNS infection episodes (*n* = 35), BDL showed a positive correlation with CRP (*r*_*s*_ = 0.48; *p* = 0.004), but not with I/T ratio, total leukocyte count, or thrombocytopenia. Subsequent linear regression analysis demonstrated a significant association between BDL and CRP (R-square 0.125; *p* = 0.04).

For *S. aureus* infection episodes (*n* = 11), no significant association was found for CRP, total leukocyte count, or thrombocytopenia with BDL. However, for the I/T ratio, there was a trend toward a strong correlation (*r*_*s*_ = 0.6; *p* = 0.09) with a positive association (adjusted R-square 0.37; *p* = 0.049). Severe complications such as mortality and shock requiring inotropy were too rare to be evaluated.

## Discussion

In this study, we evaluated a newly developed multiplex PCR for diagnosis of LOS directly in blood in a group of preterm neonates, and we explored the additional value of BDL measurement. There was no significant difference in detection rates of multiplex PCR compared with blood culture. Multiplex PCR showed a sensitivity of 77% and specificity of 81%. Both PCR and blood culture provided additional microbiological identification and thus were complementary. These findings are in line with previous reports that demonstrate the use of PCR as a rapid tool to enhance diagnosis of neonatal sepsis [8]. This study adds to the growing body of evidence that direct species identification with PCR, with results potentially available within 4 h of blood sampling as compared with 14 h of incubation, enables early initiation of targeted therapy. Twenty-five of 91 infection episodes yielded discordant results between blood culture and PCR and were mainly detections of CoNS. Interestingly, both CRP and I/T ratio were significantly lower in discordant episodes, suggesting that these neonates had less severe disease or that the detected pathogen represented contamination. The fact that two episodes with CoNS-positive blood culture and CoNS-negative PCR were regarded as noninfectious further illustrates this point. Another important explanation may be suboptimal sensitivity of PCR. Each PCR contained an equivalent of only 18 μl of blood (derived from 200 μl of blood, approximately 10% of which is used for PCR) compared with 500 μl input in blood culture. Increasing input blood volume is therefore likely to improve sensitivity. Currently, several assays are in development that allow larger input blood volumes [26, 27]. Another advantage of PCR is that it is presumably less influenced by antibiotic treatment, as reported in the literature [28].

All polymicrobial infection episodes showed discordant results between blood culture and PCR. This finding demonstrates the difficulty when diagnosing polymicrobial sepsis, such as the fact that blood culture regularly detects only the most rapidly growing microorganism. In two episodes where PCR detected both *S. aureus* and CoNS, blood culture detected only CoNS. The BDL of CoNS was > 25 times higher than that of *S. aureus*.

It is generally appreciated that blood culture serves as an imperfect reference test with both suboptimal sensitivity and specificity [29–31]. Use of a Bayesian model, which allows for evaluation of a new diagnostic test in cases of an imperfect reference test, is not possible for lack of additional reliable infection markers for this neonatal patient population [32–34].

Instead, we assessed the validity of individual discrepant blood culture PCR results (Table 3). This indicated that 2 of 11 positive blood cultures were likely false-positive results and 3 of 6 PCR results were likely true-positive results, which would increase the diagnostic utility of PCR. For two CoNS PCR-positive episodes, subsequently obtained blood samples were also positive in PCR, substantiating the true-positive result. Our quantitative measurements, the first to be reported for LOS in preterm infants, revealed often high BDL similar to measurements during meningococcal and pneumococcal disease in children [20, 21].

The magnitude of bacteremia, as assessed with quantitative culturing, is generally low but inversely related to age and highest in neonates [35]. This is in line with our results because we found high BDL that was associated with short blood culture time to positivity. The association was not as strong as one would expect, which may be partly explained by the detection of nonviable bacteria and free bacterial DNA by PCR. Furthermore, BDL correlated significantly with blood culture positivity, indicating its potential to measure the level of bacteremia.

In CoNS infection episodes, BDL was associated with CRP, indicating the potential use of BDL as a marker of severity of infection. For *S. aureus* sepsis, BDL was associated with the I/T ratio, even though only 11 episodes could be evaluated. We observed, for example, that the only infant who died of *S. aureus* sepsis had the highest BDL. These findings suggest that the course of BDL might be a useful indicator of treatment success.

This study has several limitations. First, the limited number of included infants prohibited thorough evaluation of the diagnostic performance in gram-negative sepsis and of the BDL for less prevalent pathogens. Because we recruited infants over a 4-year period, we believe we made all efforts to include a sufficient number of episodes of suspected LOS in this single-center evaluation. Second, because this assay detects DNA, it does not discriminate between viable and nonviable organisms or free and cell-associated DNA, which may have impacted the comparison of BDL with blood culture. Blood was stored at −80 °C until further processing, which could have affected the integrity of the DNA.

This is a proof-of-concept study showing that rapid bacterial species identification directly in blood is feasible in preterm neonates. Our assay could be a valuable add-on test but lacks sufficient negative predictive value to exclude sepsis. Moreover, because we performed our study in a high-risk population for which sepsis was diagnosed in 67% of episodes, it is unlikely that one test alone could exclude sepsis. Even in low-risk populations, PCR results were shown not to be sufficient to rule out sepsis [36].

The rise of antibiotic resistance and growing evidence of the adverse effects of antibiotics on gut microbiome composition, especially for neonates, further emphasizes the need for early targeted treatment [37]. Even more important, rapid diagnosis in combination with an antimicrobial stewardship program improves outcome of sepsis in the adult population [38, 39]. It is likely that this is also true for VLBW neonates, because neonatal sepsis is associated with significant morbidity and mortality [1, 40, 41].

Considering the value of multiplex PCR for diagnosis of LOS, future studies should be done to evaluate the clinical impact of this assay using an algorithmic approach that includes BDL and other infection parameters rather than PCR results only. This should be performed in a large multicenter study to ensure sufficient coverage of both gram-positive and gram-negative sepsis episodes. The ultimate aim would be to optimize antibiotic treatment by early targeted treatment and estimation of infection severity, as well as restriction of antibiotic use because unnecessary antibiotic therapy is associated with adverse outcomes [42].

## Conclusions

This study shows that multiplex PCR has fair diagnostic utility compared with blood culture, with the advantage of rapid test results. PCR provides a useful add-on test for diagnosis of LOS in preterm infants. Additional BDL determination provides a measure of infection severity that deserves to be further evaluated.

## Additional file

Additional file 1: Detection of pathogens in blood by blood culture and PCR for polymicrobial infection episodes. (DOC 51 kb)

## Abbreviations

BDL: Bacterial DNA load
CoNS: Coagulase-negative staphylococci
C_q_: quantification cycle
CRP: C-reactive protein
CSF: Cerebrospinal fluid
EAC: Extraction and amplification control
EDTA: Ethylenediaminetetraacetic acid
I/T ratio: Immature to total neutrophil ratio
LOS: Late-onset sepsis
TAT: Turnaround time
TTP: Time to positivity
VLBW: Very low birth weight
