# Supplementary material for: Evaluation of a real-time PCR assay for detection and quantification of bacterial DNA directly in blood of preterm neonates with suspected late-onset sepsis
Source: Crit Care. 2018 Apr 22;22:105. doi: 10.1186/s13054-018-2010-4 (PMC5911371; doi:10.1186/s13054-018-2010-4)
Supplement: Supplementary file 1 — Detection of pathogens in blood by blood culture and PCR for polymicrobial infection episodes. (DOC 51 kb) [file 13054_2018_2010_MOESM1_ESM.doc]

**Additional File 1**

**TABLE. Detection of Pathogens in Blood by Blood Culture and PCR for Polymicrobial Infection Episodes**

| **Patient** | **Blood Culture (+/-) /**  **PCR (+/-)** | **Blood culture**  **Microorganism** | **PCR**  **Microorganism** | **BDL**  **cfu eq/ml** | **Culture of Other Sites^a^ (Result + Microorganism)** | **CRP (mg/L)** | **Duration of Treatment (days)** | **Clinical Diagnosis** | **Comments** |
| --- | --- | --- | --- | --- | --- | --- | --- | --- | --- |
| 1 | +/+ | *Klebsiella oxytoca* + CoNS | CoNS | 104.500 | CSF (negative) | 100 | 13 | Sepsis with *Klebsiella oxytoca* and CoNS |  |
| 2 | +/+ | CoNS | *S. aureus* + CoNS | 3.190 + 88.110 | CSF (negative)  Urine (*E. coli*) | 19 | 8 | Urinary tract infection with *E. coli* |  |
| 3 | +/+ | *S. aureus* + *E. faecalis* + CoNS | *S. aureus* + CoNS | 168.850 +22.986.150 | Urine (negative) | 72 | 2 | Sepsis with *S. aureus* + *E. faecalis* + CoNS | Deceased after two days of treatment |
| 4 | +/- | *S. aureus* + CoNS | Negative | NA | CSF (negative) Pus jaw (*S. aureus*) | 50 | 16 | Sepsis with *S. aureus* |  |
| 5 | +/+ | *Lactobacillus spp.* | *S. agalactiae* + *E. faecalis* + *Klebsiella spp*. + CoNS | 440 + 3.781+ 2.750 + 1.265 | NA | 73 | 16 | NEC |  |
| 6 | +/+ | CoNS | *S. aureus* + CoNS | 165 + 51.975 | CSF (negative) Urine (negative) | 48 | 7 | Sepsis with CoNS |  |
| 7 | +/+ | *S. aureus* | CoNS | 31.130 | CSF (negative) Urine (negative) | 16 | 13 | Sepsis with *S. aureus* | Blood sample obtained after 4 hours positive in PCR for *S. aureus* |

NA: not applicable
